# Fuzzy Stochastic Petri Nets for Modeling Biological Systems with Uncertain Kinetic Parameters

**DOI:** 10.1371/journal.pone.0149674

**Published:** 2016-02-24

**Authors:** Fei Liu, Monika Heiner, Ming Yang

**Affiliations:** 1 Control and Simulation Center, Harbin Institute of Technology, Harbin, 150080 China; 2 Department of Computer Science, Brandenburg University of Technology Cottbus-Senftenberg, Cottbus, 03013 Germany; Université de Nantes, FRANCE

## Abstract

Stochastic Petri nets (SPNs) have been widely used to model randomness which is an inherent feature of biological systems. However, for many biological systems, some kinetic parameters may be uncertain due to incomplete, vague or missing kinetic data (often called fuzzy uncertainty), or naturally vary, e.g., between different individuals, experimental conditions, etc. (often called variability), which has prevented a wider application of SPNs that require accurate parameters. Considering the strength of fuzzy sets to deal with uncertain information, we apply a specific type of stochastic Petri nets, fuzzy stochastic Petri nets (FSPNs), to model and analyze biological systems with uncertain kinetic parameters. FSPNs combine SPNs and fuzzy sets, thereby taking into account both randomness and fuzziness of biological systems. For a biological system, SPNs model the randomness, while fuzzy sets model kinetic parameters with fuzzy uncertainty or variability by associating each parameter with a fuzzy number instead of a crisp real value. We introduce a simulation-based analysis method for FSPNs to explore the uncertainties of outputs resulting from the uncertainties associated with input parameters, which works equally well for bounded and unbounded models. We illustrate our approach using a yeast polarization model having an infinite state space, which shows the appropriateness of FSPNs in combination with simulation-based analysis for modeling and analyzing biological systems with uncertain information.

## Introduction

To achieve a system-level understanding of biological systems, modeling and simulation play a crucial role as they permit to represent, explain, analyze and predict the system behavior from a holistic point of view. Randomness is an inherent property of many biological processes induced by gene regulatory networks, signal transduction networks or metabolic networks [1]. Randomness may arise from the external environment, intrinsic noise or low number of molecules, and may dramatically affect the behavior of these networks [2, 3]. To deal with randomness, stochastic modeling methods have been used, e.g., chemical master equations, stochastic differential equations [1], stochastic Pi-calculus [4], stochastic process algebra [5], or stochastic Petri nets (SPNs) [6]. These approaches address the stochastic aspect of biological systems and thus describe their behavior more accurately than deterministic approaches like ordinary or partial differential equations. Among them, SPNs have recently become a promising tool. They have been widely used for modeling and analyzing stochastic biological systems.

The construction of an SPN model for a biological system basically involves two steps: building the model structure, which represents the system’s underlying biochemical reactions and their interactions, and specifying the kinetic parameters of transitions, which determine the stochastic rates of biochemical reactions. Compared with the former step, the latter one is usually more challenging. On the one hand, the information available for some kinetic parameters may be either incomplete, vague or merely qualitative (i.e., no data); in these circumstances, these kinetic parameters cannot be measured or estimated accurately (we speak of fuzzy uncertainty). On the other hand, kinetic parameters may naturally vary, e.g., between different individuals, experimental conditions, etc. (often called variability). Such fuzzy uncertainty or variability might appropriately be addressed by fuzzy sets, which permit to turn vagueness of information into mathematical representations by means of fuzzy numbers and fuzzy operations on them. However, SPNs currently cannot cope with such biological systems where both randomness and fuzziness coexist.

Therefore, in this paper we will apply a specific type of Petri nets, fuzzy stochastic Petri nets (FSPNs), to model and analyze biological systems with uncertain kinetic information. FSPNs combine SPNs and fuzzy sets, thereby taking into account both randomness and fuzziness of biological systems. For a biological system, SPNs model the randomness, while fuzzy sets model the kinetic parameters with fuzzy uncertainty or variability by associating each kinetic parameter with a fuzzy number instead of a crisp real value. With fuzzy sets, uncertain kinetic parameters of biological systems can be characterized by experts based on their knowledge or belief, presumably supported by experimental data. This approach could be useful in many areas, such as for finding suitable, i.e. biological meaningful start values or value ranges for parameter estimation, or later for the evaluation of the results obtained by help of models, respectively. Moreover, if one wants to make progress even in the light of partially missing precise data, there is no way around to use expert belief (knowledge) to obtain a sophisticated guess which is in accordance with the modeled biological reality. FSPNs allow us to consider stochastic systems which—due to incomplete or vague knowledge—have uncertain parameter values, and to analyze the uncertainties of outputs (with output referring to a moment of the stochastic process) resulting from the uncertainties associated with the input parameters. Please note, in the whole paper we assume that “uncertainty” only relates to fuzziness and has nothing to do with stochasticity. Our efficient analysis approach builds on simulation, which works equally for bounded and unbounded models, i.e., for finite or infinite state spaces; with other words, the size of the state space does not matter.

We mainly consider the following scenario where our approach may be of help to cope with the challenges on hand. For a given biological system (phenomenon), there may exist different qualitative and quantitative experimental findings (data or models), each of which may describe complementary aspects of the system. The appropriate combination of all these findings to form a more detailed model is of great significance to gain a deeper understanding of the system [7, 8]. A simplistic approach might be to convert all of these qualitative and quantitative findings into qualitative descriptions and to build a model from a merely qualitative point of view. But it becomes more challenging the other way around. In this paper, we aim to use our approach for integrating qualitative experimental findings into a quantitative model and to explore the system to be studied from the quantitative point of view. This may be achieved by describing qualitative or semi-quantitative findings using fuzzy sets.

The main contributions of this paper are as follows.

- We apply a new modeling formalism, FSPNs, to cope simultaneously with both randomness and fuzziness of biological systems by combining SPNs and fuzzy sets. Thus, a biological system can be analyzed even if it has some parameters that are unknown or not precisely estimated.
- We present a simulation-based analysis method for FSPNs, and thus we can analyze the uncertainties of outputs caused by the uncertainties associated with the input parameters for both bounded or unbounded models independently of the size of the state space.
- Our approach permits to explore both the transient and steady state behavior, and can be easily varied to cope with different types of fuzzy numbers.

This paper is structured as follows. After a brief review of related work in Section 2, we describe fuzzy stochastic Petri nets in Section 3, followed by a simulation-based analysis method for FSPNs in Section 4. After that, we use an example of the yeast polarization model to illustrate our method in Section 5, followed by the discussions and conclusions in Section 6.

## Related work

### Stochastic Petri nets for modeling biological systems

Stochastic modeling is able to capture the randomness and fine grain behavior of biological systems which are not appropriately described by deterministic methods confining themselves to the averaged behavior. SPNs have been widely used for modeling stochastic biological systems since Gross et al. first applied stochastic Petri nets to molecular biology [9]. For example, Srivastava et al. [10] modeled the Escherichia coli stress circuit and performed stochastic kinetic analysis using SPNs. Marwan et al. [11] reconstructed the regulatory network controling the commitment and sporulation in *Physarum polycephalum* by use of hierarchically structured SPNs. Mura et al. [12] described the cell cycle in yeast with SPNs, and Lamprecht et al. [13] develop an SPN model for Ca^2+^ release sites consisting of a number of intracellular Ca^2+^ channels that exhibit stochastic Ca^2+^ excitability. Marwan et al. [14] investigated phosphate regulation in enteric bacteria by use of SPNs, while Castaldi et al. [15] gave an SPN model of the tissue factor induced coagulation cascade. Colored SPNs, an extension of SPNs by data structures as known from programming languages, have been used by Liu et al. [16] to discuss the modeling of membrane systems deploying an example of the virus infection, and by Pârvu et al. [17] to explore the stochasticity of bacterial colony growth in space with phase variable genes. See [6, 18, 19] and references therein for more applications. Like other quantitative modeling methods, SPNs rely on accurate kinetic data; in any case, the results shall be very carefully cross-validated.

### Petri net based parameter estimation

Parameter estimation plays an important role in the construction of quantitative models. The aim is to tune the kinetic parameters to fit simulation results to in vivo/vitro experiment observations. A lot of work has been done to address this challenging problem, some of them within a Petri net framework. For example, Shaw et al. [20] proposed an automatic parameterization method for SPN models of biological networks using a distributed genetic algorithm, and illustrated its application using the stress response pathway in the bacterium E. coli. Koh et al. [21] gave a decompositional approach to parameter estimation in pathway models using hybrid functional Petri nets (HFPN). It exploits the structure of a large pathway model to decompose it into smaller components and then estimates the parameters independently. Donaldson and Gilbert [22] characterize the desired behavior of SPN models by properties expressed in a Probabilistic Linear-time Temporal Logic with numerical constraints and combine Monte Carlo Model Checking with genetic algorithms to alter the parameters in the SPN model. Li et al. [23] utilized a data assimilation framework and model checking to estimate kinetic parameters of biological pathways, which were described by hybrid functional Petri net with extension (HFPNe).

In contrast, our approach in this paper differs from parameter estimation in the following way. Parameter estimation means to tune parameters and find crisp values to fit the simulation results to in vivo/vitro experiment observations, i.e., removing parameter uncertainties, while we want to derive the uncertainties of outputs which are caused by uncertain input parameters, i.e., keeping parameter uncertainties.

### Fuzzy logic for modeling biological systems

Fuzzy logic is able to appropriately capture systems with only qualitative or incomplete quantitative information by describing the dynamic behavior of a system with a set of fuzzy rules. It has been applied for the modeling of a number of biological systems, see e.g., [24]. Fuzzy Petri nets, combining fuzzy logic and (qualitative) Petri nets, facilitate the use of fuzzy logic by help of the graphical representation coming with Petri nets. They have occasionally been used for biological modeling, see, e.g., [25, 26]. The advantage of fuzzy logic or fuzzy Petri nets is that they can be deployed to model biological systems if the kinetic data are incomplete or even unavailable; the disadvantage is that they are usually used for constructing qualitative models, rather than quantitative ones.

To overcome this, researchers started to combine quantitative Petri nets with fuzzy logic. For example, Valette et al. [27] introduced a class of fuzzy time Petri nets by associating transitions with fuzzy enabling durations. Ding et al. [28] gave a class of fuzzy timed Petri nets in which a fuzzy number is associated with a transition. Tüysüz et al. [29] combined stochastic Petri nets with fuzzy sets and applied it to the modeling of a flexible manufacturing cell with fuzzy parameters. But to the best of our knowledge, there are so far no applications of these fuzzy quantitative Petri nets to the modeling of biological systems.

In this paper, we combine SPNs and fuzzy logic in order to quantitatively model biological systems with uncertain kinetic parameters. We will use a similar way to associate the rate of a stochastic transition with a fuzzy number as in [29]. However, in [29], they analytically determine the steady state distribution of an SPN model by generating the corresponding continuous time Markov chain (CTMC). As this approach requires a finite state space, they only can deal with bounded Petri nets. But, there are many biological models that are unbounded. Unbounded SPNs have an infinite state space, thus the CTMC cannot be exhaustively constructed. Consequently, all numerical approaches requiring a closed representation of the CTMC do not work. Even if the model is bounded, the size of the state space often exceeds the computational resources required for numerical analyses; see [30] for details.

Therefore, in this paper we will adopt a simulation approach to tackle both bounded and unbounded models in order to overcome the limitations of the method developed in [29]. Moreover, our approach immediately reveals both the transient and steady state behavior, and can be easily varied to cope with different types of fuzzy numbers.

## Fuzzy stochastic Petri nets

### Stochastic Petri nets

**Fig 1:**
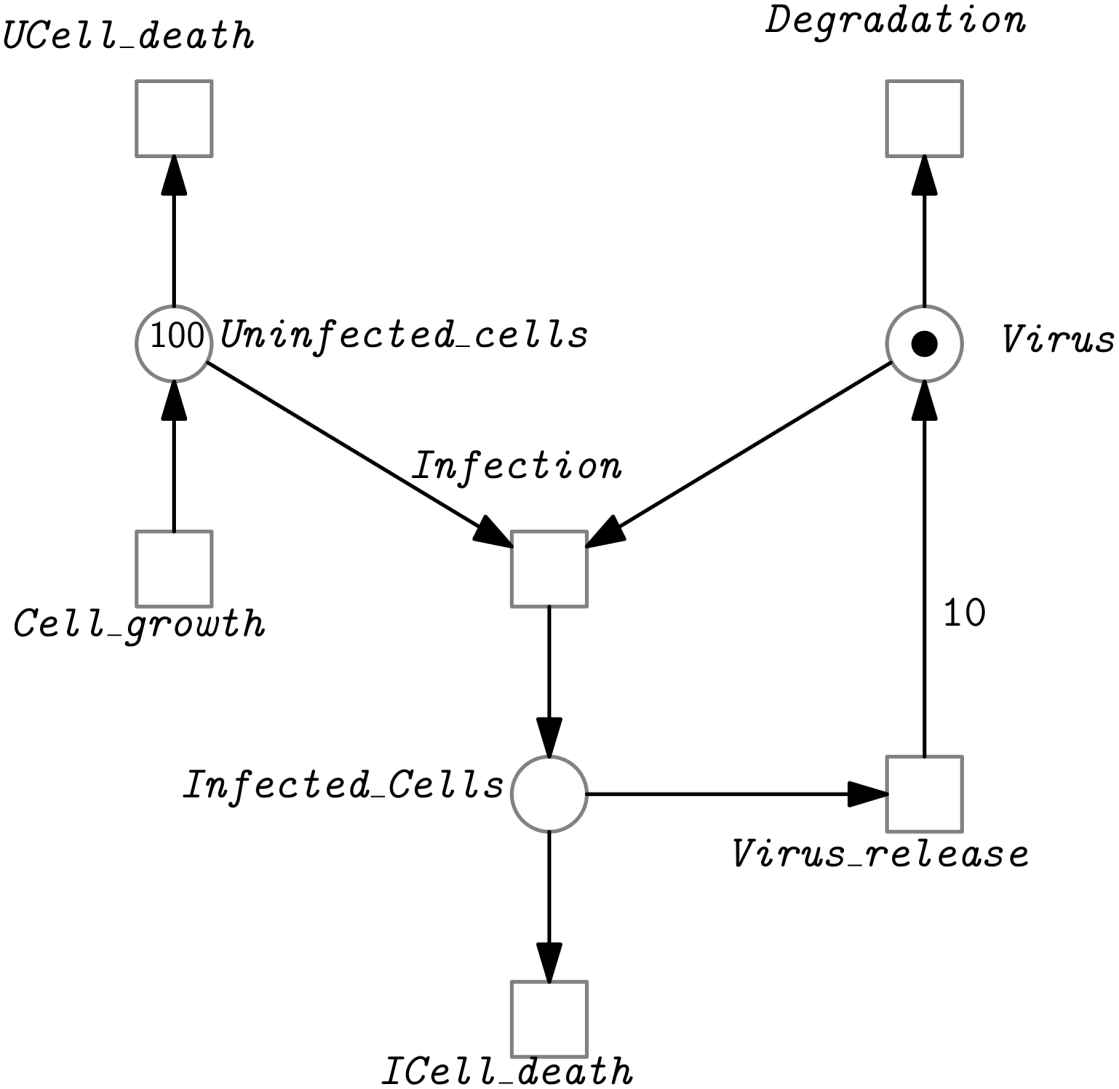
A Petri net model for the infection of healthy cells by a virus [34]. Cells grow or die. The virus may enter a healthy cell (UCell) and infect it (ICell). Then the virus starts the replication of itself and more viruses are released (note the arc weight of 10). Besides, infected cells may die and viruses may degrade.

Petri nets [31, 32] (see Fig 1 for an example) are weighted, directed, bipartite multigraphs, which consist of places, transitions and arcs. In biological models, places may represent chemical species or any kind of compounds, e.g., genes, mRNA, proteins or protein complexes, while transitions represent any kind of chemical reactions, e.g. association, disassociation, translation, translocation or transcription [33]. For a chemical reaction, its precursors correspond to the preplaces of a transition, while products correspond to its postplaces. The preplaces (postplaces) of a transition are also called the input (output) places of the transition. The arcs connect places with transitions, or transitions with places. The weight of an arc indicates its multiplicity, reflecting e.g., stoichiometries of a chemical reaction. The weight 1 is usually not explicitly labeled. A place may contain an arbitrary (natural) number of tokens, represented as black dots if the number of tokens is smaller, or a natural number if it is larger. A specific distribution of tokens over all places of a Petri net is called a marking, which represents a state of the Petri net. The formal definition of a (qualitative) Petri net is given in the following.

A Petri net PN is a five-tuple *N* = <*P*, *T*, *F*, *f*, *M*_0_>, where

- *P* is a finite, non-empty set of places.
- *T* is a finite, non-empty set of transitions.
- *F* ⊆ (*P* × *T*)∪(*T* × *P*) is a finite set of directed arcs.
- $f:F\to N^{+}$ is a function that assigns a positive integer to each arc *a* ∈ *F*.
- $M_{0}:P\to N_{0}$ gives the initial marking, which assigns a non-negative integer to each place *p* ∈ *P*.

A transition is called enabled if each of its preplaces contains tokens whose number is greater than or equal to the weight of the corresponding arcs. If a transition is enabled, it may fire. The firing of a transition moves tokens from its preplaces to its postplaces according to the weights of the corresponding arcs, updating the current marking to a new reachable one. The repeated firing of transitions establishes the behavior of a net. The set of markings reachable from the initial marking form the state space of the net. These reachable markings and transitions between them constitute the reachability graph of the net.

Stochastic Petri nets are a popular extension of Petri nets [6, 35], and more specifically can be considered as an extension of Time Petri nets (TPN) [36]. In TPN, deterministic firing delays are associated to transitions. Once a transition is enabled, it may fire after the elapsing of the associated deterministic delay time, provided it did not loose the concession to fire while waiting. In SPN, these firing delays are considered as random variables with a negative exponential probability distribution function (PDF). For this purpose, a stochastic hazard function *h*(*t*, *θ*) (or firing rate) is assigned to each transition *t* ∈ *T*, whereby

$$\begin{array}{c} h\left(t,\theta \right):N_{0}^{{|}^{•}t|}\to R^{+}. \end{array}$$ (1)

*θ* is a rate constant (also called kinetic parameter) which serves as parameter for the PDF, $R^{+}$ is the set of all non-negative real numbers, and ^•^*t* denotes the preplaces of *t*. A classical type of hazard functions are the Gillespie propensities: *h*(*t*, *θ*) = *θ* ⋅ #{reactant combinations}. The stochastic hazard function *h*(*t*, *θ*) relates to a random variable *Y*_*t*_, defined by the following exponential probability distribution (written as *Y*_*t*_ ∼ *Exp*(*h*(*t*, *θ*))):

$$\begin{array}{c} F_{Y_{t}}\left(\tau \right)=1-e^{-h(t,\theta )\cdot \tau },\tau \geq 0\;. \end{array}$$ (2)

The semantics of a stochastic Petri net is defined by a continuous time Markov chain (CTMC), a class of stochastic processes, which is constructed from the reachability graph of the underlying qualitative Petri net by labeling the arcs between the states (markings) with the corresponding transition rates, i.e., all states are still reachable; for more details see [6].

Formally, the stochastic process underlying a stochastic Petri net can be described as a collection of random variables indexed by means of time indices *τ* from a time set *T*′, given a probability space (*Ω*, *Σ*, *Pr*), where *Ω* is a sample space, *Σ* is a *σ*-algebra of subsets of *Ω* and *Pr* is a probability measure, that is,

$$\begin{array}{c} \left\{X_{\tau }:\tau \in T^{{}^{\prime }}\right\} \end{array}$$ (3)

where each *X*_*τ*_ (where *X* is the marking of the stochastic Petri net) is a random variable on *Ω*. See e.g., [37] for more details.

**Fig 2:**
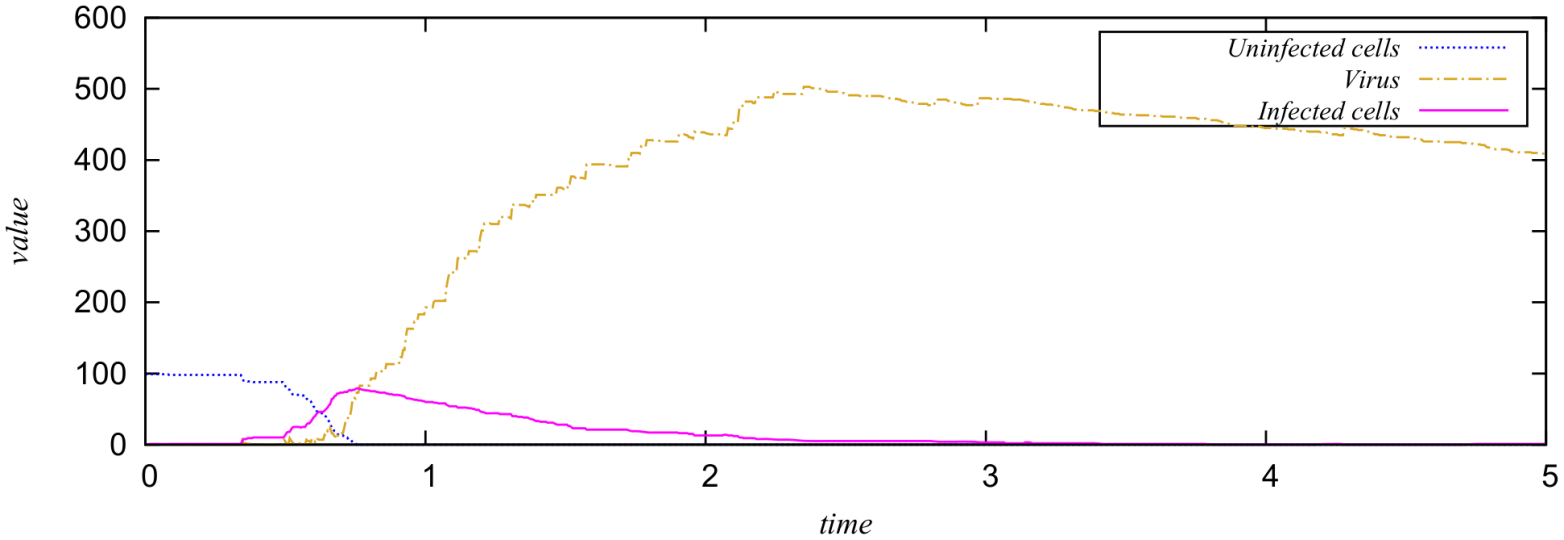
A stochastic simulation plot for the virus infection SPN model shown in Fig 1 with the rate functions given in Table 1.

For example, if we assign the rate functions given in Table 1 to the model in Fig 1, then we obtain an SPN model; see Fig 2 for a stochastic simulation plot. The model assumes infinite cell growth (its corresponding transition does not have a precondition). Thus, the corresponding CTMC is infinite. The averaged amount of uninfected cells depends on the ratio of the firing rates of the pre- and posttransitions.

**Table 1:** Rate functions for the virus infection SPN model [34]. Here, the place names like Uninfected_cells refer to the markings of these places.

transition *t*	rate function *h*(*t*, *θ*)	kinetic parameter *θ*
*Cell_growth*	*θ*_1_	1.0
*UCell_death*	*θ*_2_*Uninfected_cells	0.1
*Infection*	*θ*_3_*Uninfected_cells*Virus	1.0
*Virus_release*	*θ*_4_*Infected_Cells	1.0
*ICell_death*	*θ*_5_*Infected_cells	0.5
*Degradation*	*θ*_6_*Virus	0.1

### Fuzzy sets

Fuzzy theory was introduced by Zadeh [38] to deal with uncertainty associated with imprecision or vagueness rather than with randomness. A fuzzy set $\tilde{\xi }$ on a universal set X is defined by its membership function

$$\begin{array}{c} \mu_{\tilde{\xi }}:X\to \left[0,1\right], \end{array}$$ (4)

which assigns to each element x∈X a real value $\mu_{\tilde{\xi }}\left(x\right)$ in [0, 1]. The support of a fuzzy set $\tilde{\xi }$ is the crisp subset of all elements in X with non-zero membership degree, i.e.,

$$\begin{array}{c} supp\left(\tilde{\xi }\right)=\left\{x|\mu_{\tilde{\xi }}\left(x\right)>0,x\in X\right\}. \end{array}$$ (5)

The core of a fuzzy set $\tilde{\xi }$ is the crisp subset of all elements in X with membership degree equal to 1, i.e.,

$$\begin{array}{c} core\left(\tilde{\xi }\right)=\left\{x|\mu_{\tilde{\xi }}\left(x\right)=1,x\in X\right\}. \end{array}$$ (6)

The *α*-cut of a fuzzy set $\tilde{\xi }$ for a level *α* ∈ [0, 1] is the crisp subset of X, containing all the elements whose membership degree is greater than or equal to a given *α*, i.e.,

$$\begin{array}{c} {\tilde{\xi }}_{\alpha }=\left\{x|\mu_{\tilde{\xi }}\left(x\right)\geq \alpha ,x\in X,\alpha \in \left[0,1\right]\right\}. \end{array}$$ (7)

**Fig 3:**
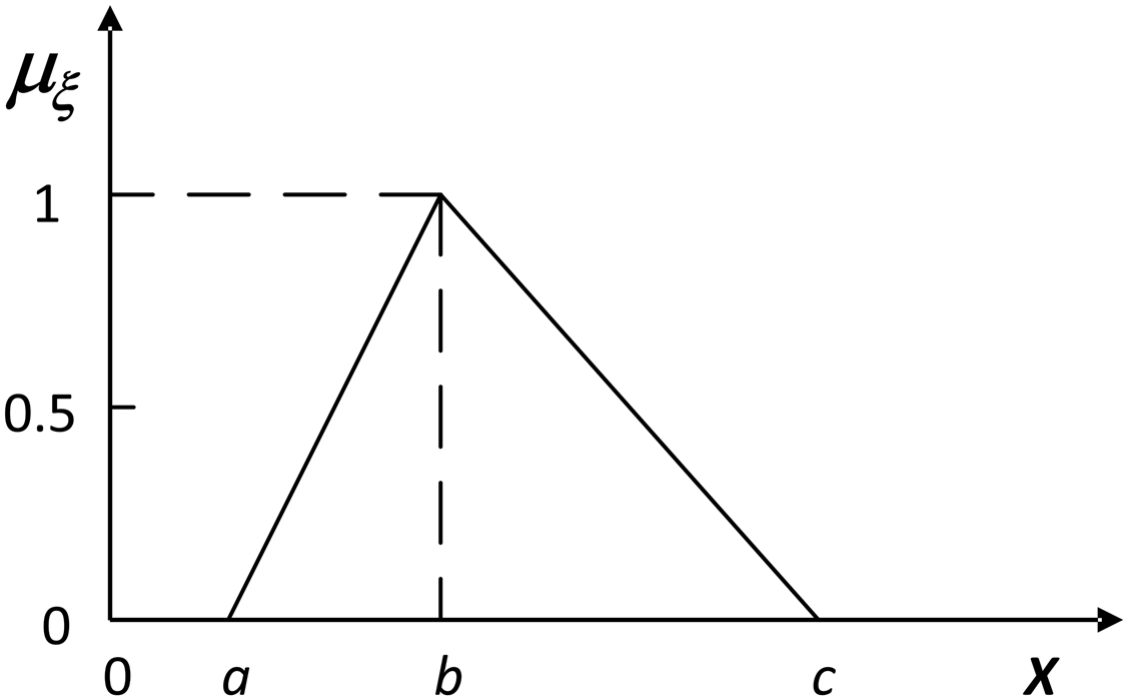
A triangular fuzzy number.

A fuzzy number is a special convex (that is, each of its *α*-cuts is a convex set) normalized fuzzy set defined on the real set R. Among different fuzzy numbers, triangular and trapezoidal shapes are widely used. For example, a triangular fuzzy number, denoted by $\tilde{\xi }=\left(a,b,c\right)$, *a* ≤ *b* ≤ *c*, is defined as follows (see Fig 3):

$$\begin{array}{c} \mu_{\tilde{\xi }}\left(x\right)=\left\{\begin{array}{cc} 0 & \;\text{if}\;x\leq a,\\ \frac{x-a}{b-a} & \;\text{if}\;a\leq x\leq b,\\ \frac{c-x}{c-b} & \;\text{if}\;b\leq x\leq c,\\ 0 & \;\text{if}\;x\geq c, \end{array}\right. \end{array}$$ (8)

and its *α*-cut is simply ${\tilde{\xi }}_{\alpha }=\left[a+\alpha \left(b-a\right),c-\alpha \left(c-b\right)\right]$, for any *α* ∈ [0, 1]. We then denote by Γ the set of fuzzy numbers whose lower bound is greater than 0.

### Fuzzy stochastic Petri nets

Fuzzy stochastic Petri nets are an extension of SPNs by associating each transition *t* ∈ *T* with a stochastic hazard function *h*(*t*, *θ*), where

$$\begin{array}{c} h\left(t,\theta \right):N_{0}^{{|}^{•}t|}\to R^{+}\cup \Gamma , \end{array}$$ (9)

and the kinetic parameter *θ* is described by either a fuzzy number in Γ or a real value in $R^{+}$. Please note that in our definition, we allow *θ* to be a real value if it is measured accurately, or a fuzzy number if it is uncertain or varies. When each fuzzy number *θ* in *h*(*t*, *θ*) is defuzzified or sampled to a crisp value, the FSPN turns into an SPN.

Formally, a fuzzy stochastic Petri net is a six-tuple *N* = <*P*, *T*, *F*, *f*, *v*, *M*_0_>, where

- <*P*, *T*, *F*, *f*, *M*_0_> is a Petri net.
- *v*: *T* → *H* is a function that assigns a stochastic hazard function *h*(*t*, *θ*) to each transition *t* ∈ *T*, whereby *H*: = ⋃_*t* ∈ *T*_{*h*(*t*, *θ*)} is the set of all stochastic hazard functions, *v*(*t*) = *h*(*t*, *θ*) for all transitions *t* ∈ *T*, and *h*(*t*, *θ*) is defined by Eq 9.

For example, if we assign the rate functions given in Table 2 to the model in Fig 1, then we obtain an FSPN model.

**Table 2:** Rate functions for the virus infection FSPN model. Here, the place names like Uninfected_cells refer to the markings of these places.

transition *t*	rate function *h*(*t*, *θ*)	kinetic parameter *θ*
*Cell_growth*	*θ*_1_	(0.9, 1, 1.4)
*UCell_death*	*θ*_2_*Uninfected_cells	(0.05, 0.1, 0.2)
*Infection*	*θ*_3_*Uninfected_cells*Virus	(0.9, 1, 1.2)
*Virus_release*	*θ*_4_*Infected_Cells	(0.9, 1, 1.2)
*ICell_death*	*θ*_5_*Infected_cells	(0.4, 0.5, 0.6)
*Degradation*	*θ*_6_*Virus	(0.05, 0.1, 0.15)

In contrast with the probability measure and random variables in the probability theory which allow us to deal with stochasticity, the possibility measure and fuzzy variables in the possibility theory [39] are deployed to describe the fuzzy uncertainty [40]. Suppose (Θ, *P*(Θ), *Pos*) is a possibility space, where Θ is the sample space, *P*(Θ) is the power set of Θ, and *Pos* is the possibility measure on Θ. A mapping from Θ to the real set R defines a fuzzy variable. For example, a fuzzy number is a fuzzy variable. A random fuzzy variable *ξ* is defined as a mapping from the possibility space (Θ, *P*(Θ), *Pos*) to a set of random variables [41]. For example, suppose *ξ* ∼ *Exp*(*λ*), where *λ* is a fuzzy variable; then *ξ* is a random fuzzy variable. For each *θ* ∈ Θ, if the expected value *E*[*ξ*(*θ*)] is finite, then *E*[*ξ*(*θ*)] is also a fuzzy variable in (Θ, *P*(Θ), *Pos*).

Further, for a fuzzy stochastic Petri net, each *X*_*τ*_ in Eq 3 can be considered as a random fuzzy variable. Accordingly, a moment of *X*_*τ*_ like *E*[*X*_*τ*_] can be considered as a fuzzy variable (fuzzy moment). Therefore, the semantics of a fuzzy stochastic Petri net can be regarded as a fuzzy stochastic process (that is a mapping from the possibility space to the set of random stochastic processes) entirely determined by its “fuzzy probability distribution” (of the family of random fuzzy variables), which can be described by the set of fuzzy moments like *E*[*X*_*τ*_].

We can further define an observation function or performance measure *f*(*X*_*τ*_) based on *X*_*τ*_, and thus a moment of *f*(*X*_*τ*_) like *E*[*f*(*X*_*τ*_)]is also a fuzzy variable, with which we can analyze a fuzzy stochastic Petri net. Therefore, for a fuzzy stochastic Petri net, according to the membership functions of fuzzy input parameters, we obtain the membership functions of outputs, as each output can be considered as a fuzzy variable.

FSPN provides an alternative approach to immediately study those kinds of systems where precise values for some parameters cannot be obtained for the time being. Thus, instead of idle waiting for complete (crisp) parameters, the fuzzy set approach permits to make progress building on expert belief. As to how to build a FSPN model, parameter estimation should always be the first choice to possibly obtain the precise values of most parameters, and fuzzy values are assigned by professionals to those parameters and situations only where experimental data are not available yet. Besides, to determine the values of uncertain or unknown kinetic parameters, we could adopt the following scheme. The professional experts of the field may use their special insight knowledge of the system to be studied or the available parameter estimation results, to specify the pessimistic value, the most possible value and the optimistic value for an uncertain or unknown parameter, which then form a fuzzy number for this parameter. Experts in this context are usually those most familiar with the modeled system or pathway under consideration.

## Simulation and analysis

SPN models that are not bounded induce an infinite state space, and thus an infinite CTMC, which prevents the use of any analysis technique building on a finite CTMC representation. Simulation-based behavior exploration is the only possible approach, if the state space is too huge to be analyzed numerically or even infinite. In this case, the size of the state space one can reach depends on the number of simulations that are done and of course on the length of the simulation traces. In this paper, we will overcome this problem by a simulation-based analysis method. The general idea of this method is as follows.

According to Zadeh’s extension principle [38], a fuzzy variable (number) can be represented as a union of its *α*-cuts. Therefore, if we decompose all fuzzy parameters into its *α*-cuts, and then run stochastic simulations at each *α* level, we obtain the *α*-cut for each output of interest. We then compose all the *α*-cuts and obtain the membership function for each output, which reflects the effect of the uncertainties of the input parameters. The whole procedure is given in Algorithm 1, which consists of the following three steps.

**Algorithm 1** Simulation algorithm for an FSPN model.

**Input:** An FSPN model

**Output:** Membership functions of the performance measures of interest

1: **for** each *α* level *α*_*j*_, *j* = 1, 2, …, *J* **do**

2:  **for** each fuzzy number ${\tilde{\theta }}_{i}$, *i* = 1, 2, …, *I* **do**

3:   Obtain its *α*-cut, represented as ${\left({\tilde{\theta }}_{i}\right)}_{\alpha_{j}}=\left[L_{i}^{j},U_{i}^{j}\right]$;

4:   Discretize each *α*-cut $\left[L_{i}^{j},U_{i}^{j}\right]$ and obtain crisp values for each parameter;

5:  **end for**

6:  **for** each combination of values for all parameters **do**

7:   Run stochastic simulations for *M* replications;

8:   **for** each performance measure *Y*_*m*_, *m* = 1, 2, …, *M* **do**

9:    Compute the sample mean ${\bar{Y}}_{m}$;

10:    Compose all the *α*-cuts of *Y*_*m*_ to obtain its membership function;

11:   **end for**

12:  **end for**

13: **end for**

**Fig 4:**
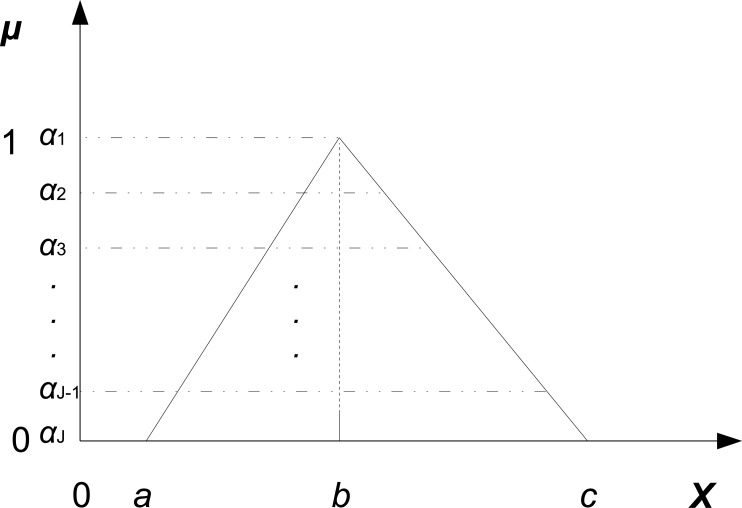
Decomposition of a fuzzy number into its *α*-cuts.

(1) For each fuzzy number ${\tilde{\theta }}_{i}$, *i* = 1, 2, …, *I*, decompose its membership function into some of its *α*-cuts using the same step size, denoted, e.g., by *α*_*j*_, *j* = 1, 2, …, *J*, where *J* is the number of the considered *α* levels; see Fig 4 for a demo. The step size of the *α* levels should be determined with great care according to the given problem. A smaller step may achieve a precise membership function for a given output, but may also cause a significant increase in computational costs. The *α*-cut for each fuzzy number ${\tilde{\theta }}_{i}$ can be represented as ${\left({\tilde{\theta }}_{i}\right)}_{\alpha_{j}}=\left[L_{i}^{j},U_{i}^{j}\right]$. After that we discretize each *α*-cut $\left[L_{i}^{j},U_{i}^{j}\right]$ using different kinds of sampling methods, such as Latin Hypercube sampling (LHS), and obtain a set of crisp values for each parameter. We denote by *K* the number of the crisp values after discretization. To obtain crisp values (points), a simple division of the whole *α*-cut into a number of smaller equally sized intervals is usually enough. At each *α* level *α*_*j*_, we obtain all combinations of values for all parameters. Please note that this approach can be applied regardless of the form of fuzzy numbers. But, for sake of simplicity, we have chosen in the following a rather simple version of fuzzy numbers, which can effortlessly be replaced by any more sophisticated fuzzy number scheme.
(2) For each combination, we apply Gillespie’s stochastic simulation algorithm (SSA) [42] to produce simulation results, and then analyze some key performance measures (or outputs), denoted by *Y*_*m*_, *m* = 1, 2, …, *M*, where *M* is the number of performance measures of interest. For example, a performance measure could be the average number of molecules of a species at some time point.

For a transient simulation (i.e., a simulation which aims to study the transient behavior), we usually run a number of independent and identically distributed (i.i.d.) replications to compute the sample mean as the point estimator of the expectation for a performance measure. For example,

$$\begin{array}{c} {\bar{Y}}_{m}=\frac{1}{N}\sum_{n=1}^{N}{\left(Y_{m}\right)}_{n}, \end{array}$$ (10)

where (*Y*_*m*_)_*n*_ is the output data at simulation replication *n*, and *N* is the number of replications. Further, we can compute a confidence interval for the expectation; see, e.g., [43] for more details about transient simulation analysis. A crucial point here is to determine the required amount of simulation replications (or samples) in order to achieve a reliable estimation of the measure of interest. For this, we can use the idea of the confidence interval as described in [44]. The confidence interval relates to a confidence level, which usually has values of 90%, 95%, or 99%. For example, choosing the confidence level of 95% and the accuracy of 10^−2^, we need to perform—according to Eq 4 in [44]—38,000 stochastic simulation replications. As this is usually computationally intensive due to the numerous numerical simulations, we have to decide on an appropriate number of simulation runs by choosing an acceptable confidence level and accuracy according to e.g., [44]. That is, sometimes, we have to choose a small confidence level (e.g., 90%) and low accuracy (e.g., 10^−1^) in order to decrease the number of simulation runs.

For a steady state simulation (i.e., a simulation which aims to study the steady state behavior), the number of samples required to estimate a performance measure can be obtained using a single long simulation run by viewing the estimator as the long-run average level. But we have to ensure that the simulation reaches a stationary state and the effect of the initial values has been overcome. In particular, given the simulation observations (*Y*_*m*_)_*i*_, *i* = 1, 2, …, *L*, where *L* is the length of a simulation run, we could use

$$\begin{array}{c} {\bar{Y}}_{m}^{l}=\frac{1}{L-l}\sum_{i=l+1}^{L}{\left(Y_{m}\right)}_{i}, \end{array}$$ (11)

to estimate the steady state mean of a performance measure. Here we adopt the most commonly used method given in [45] for eliminating the bias caused by the initial conditions, that is, identifying an index *l* from which the warmup period finishes and truncating the observations before the index *l*. See, e.g., [43] for more details about steady state simulation.

(3) The sample mean ${\bar{Y}}_{m}$ for each combination consists of the *α*-cut of a measure *Y*_*m*_ at level *α*_*j*_. In this way, we obtain all the *α*-cuts of a measure *Y*_*m*_.

**Fig 5:**
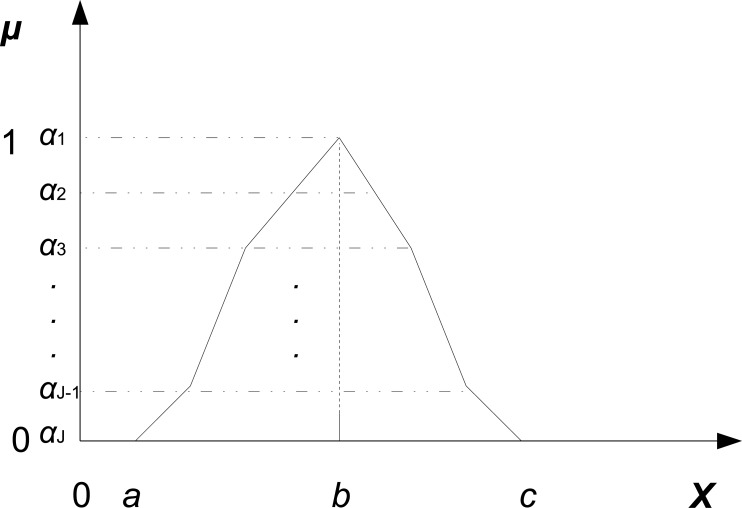
Composition of the *α*-cuts to obtain a fuzzy number.

According to Zadeh’s extension principle [38], saying that a fuzzy variable can be represented as a union of its *α*-cuts, we can compose all the *α*-cuts of a measure *Y*_*m*_ to obtain its membership function, which reflects the effect of the uncertainties of parameters on the uncertainties of the measure; see Fig 5 for a demo.

**Reproducibility.** We use an extended version of Snoopy [46], a unifying, platform-independent and easily extensible Petri net tool, for modeling our FSPNs. Then, we deploy Matlab [47] for performing fuzzy and stochastic simulations by implementing the algorithm described above and for drawing all the plots given in this paper. The interested reader can download the extended version of Snoopy (Windows OS only), the Matlab code, and all the models used in the paper from http://dx.doi.org/10.6084/m9.figshare.1451230, which then will permit to reproduce our simulation results.

## Example

We now illustrate our approach using the yeast polarization model describing the pheromone-induced G-protein cycle in *Saccharomyces cerevisiae* [48, 49], which consists of the following eight reactions.

$$\begin{array}{cc} r_{1}: & \emptyset \overset{k_{1}}{\to }R\\ r_{2}: & R\overset{k_{2}}{\to }\emptyset \\ r_{3}: & L+R\overset{k_{3}}{\to }L+RL\\ r_{4}: & RL\overset{k_{4}}{\to }R\\ r_{5}: & RL+G\overset{k_{5}}{\to }G_{a}+G_{bg}\\ r_{6}: & G_{a}\overset{k_{6}}{\to }G_{d}\\ r_{7}: & G_{d}+G_{bg}\overset{k_{7}}{\to }G\\ r_{8}: & \emptyset \overset{k_{8}}{\to }RL \end{array}$$

In this model, *R*, *L*, and *RL* represent the pheromone receptors, ligands, and receptor-ligand complexes, respectively. *G* represents the G-protein, and *G*_*a*_, *G*_*d*_ and *G*_*bg*_ its three separate units. The ligands *L* bind with the receptors *R* to form complexes *RL*, which activate the G-proteins *G* to separate its two subunits *G*_*a*_ and *G*_*bg*_. *G*_*a*_, acting as an autophosphotase, can be dephosphorylated to *G*_*d*_, which then rebinds with *G*_*bg*_ to go back to *G*. This completes the whole pheromone-induced G-protein cycle.

**Fig 6:**
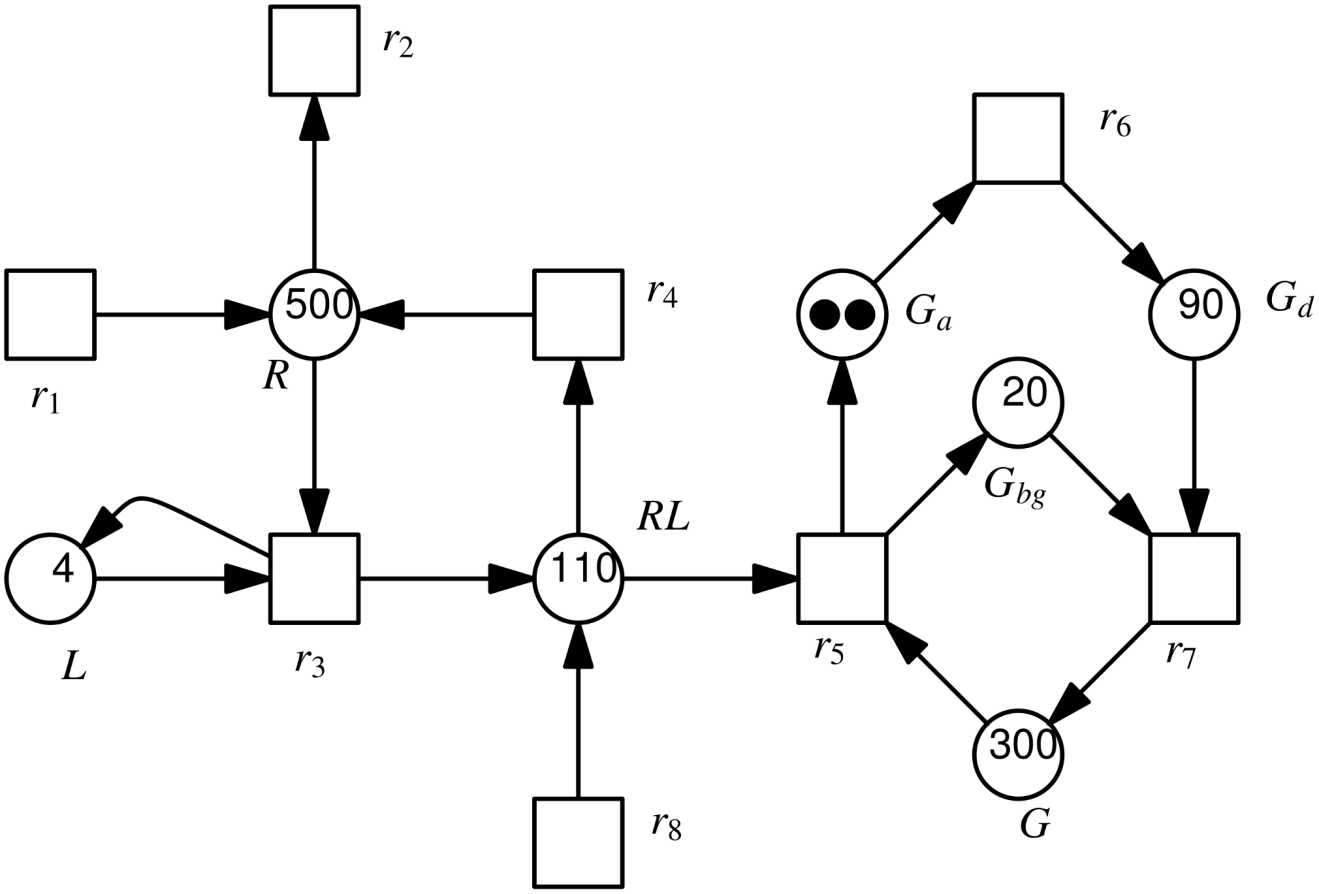
An FSPN model for yeast polarization based on the eight reactions given above.

We create an FSPN model of the G-protein cycle based on the reactions given above, which is shown in Fig 6. Each species is modeled as a place and each reaction as a transition, and thus the FSPN model has 7 places and 8 transitions. That is, we can write the place set as *P* = (*L*, *R*, *RL*, *G*, *G*_*bg*_, *G*_*a*_, *G*_*d*_), and the transition set as *T* = (*r*_1_, *r*_2_, *r*_3_, *r*_4_, *r*_5_, *r*_6_, *r*_7_, *r*_8_). Besides, we denote by *k* = (*k*_1_, *k*_2_, *k*_3_, *k*_4_, *k*_5_, *k*_6_, *k*_7_, *k*_8_) the kinetic parameter set.

We start our analysis by determining some structural properties of the FSPN model using the Petri net analysis tool Charlie [50] (see [6] for the technical notions used in the following brief discussion). It turns out that this model is unbounded due to the two input transitions *r*_1_, *r*_8_. So its infinite CTMC cannot be constructed, but we can use our simulation-based method to explore its behavior. We further compute the invariants of the Petri nets, and obtain three minimal semi-positive place invariant, (*L*), (*G* + *G*_*bg*_), and (*G* + *G*_*a*_ + *G*_*d*_), leaving *R* and *RL* as the places where the number of tokens is structurally unbounded. As *L* establishes a place invariant on its own, the token number on *L* will never change and remains during the execution always the same as at the initial marking. Thus, we can neglect it during the following analysis. Computing the transition invariants reveals that all reactions may contribute to the steady state behavior (technically speaking, the Petri net is covered with transition invariants). Finally, the so-called Siphon-Trap Property (STP) holds, which ensures for the given net structure (Extended Simple) that the Petri net is live, meaning all reactions will occur forever.

**Fig 7:**
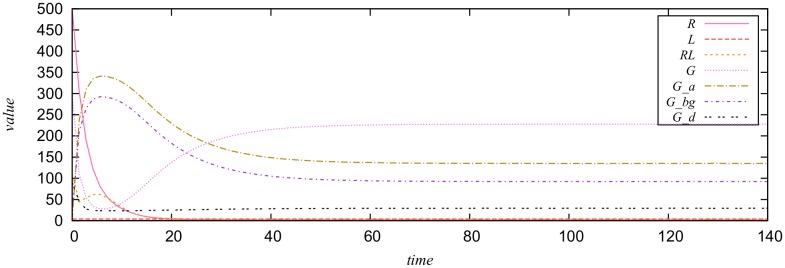
A stochastic plot of the yeast polarization model.

We set the initial marking to *M*_0_(*P*) = (4,500, 110, 300, 20, 2, 90) and use the parameter values *k* = (0.38, 0.04, 0.082, 0.12, 0.021, 0.1, 0.005, 13.21), both taken from [51]. In this case, the model is an SPN model, and we run stochastic simulation for 38,000 replications to obtain an average simulation trace, which is given in Fig 7. The plot shows that each species reaches a steady state in this example. But note that the convergence of expected values does not necessarily mean that the stochastic process reaches a steady state.

We then explore how the uncertainties of kinetic parameters affect the outputs using the simulation-based method introduced above. For each fuzzy parameter, we consider 11 *α* levels, i.e., 0.0, 0.1, …, 1.0, and equally discretize each *α*-cut into 11 points. For each combination of parameter values, we run a long simulation by setting the simulation end time to 38,000 and compute the steady state mean for each performance measure of interest using Eq 11. The reason for choosing this simulation end time is as follows. For transient simulation, 38,000 simulation runs offer 38,000 samples to compute the sample mean according to Eq 10, which results in the confidence level of about 95% and the accuracy of 10^−2^ according to Eq 4 in [44]. If we set the simulation end time to 38,000 and sample a trace every single simulation time unit, we also obtain 38,000 samples to compute a sample mean, which results in a similar confidence level and accuracy as when doing 38,000 runs for transient simulation.

**Fig 8:**
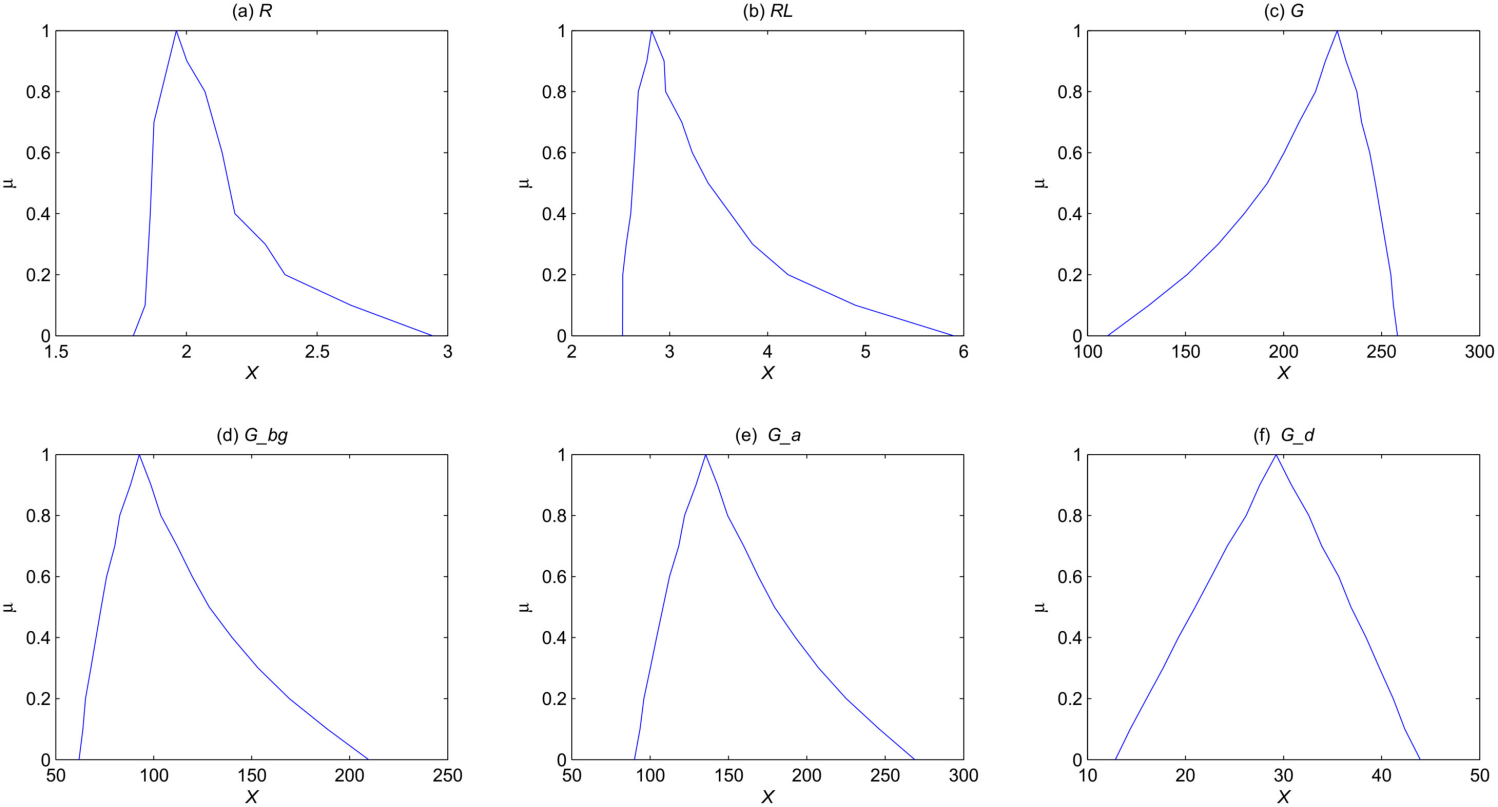
The membership function of the steady state mean for each species of the yeast polarization model in the setting of one fuzzy parameter *k*_6_ = (0.05, 0.1, 0.15).

**Fig 9:**
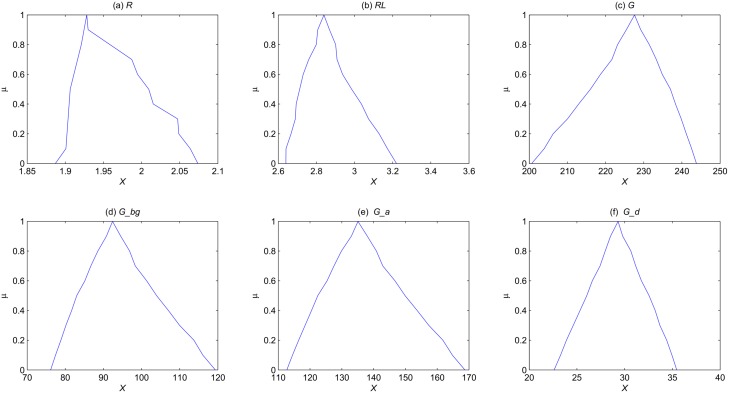
The membership function of the steady mean for each species of the yeast polarization model in the setting of one fuzzy parameter *k*_6_ = (0.08, 0.1, 0.12).

First, we consider only one fuzzy parameter *k*_6_ = (0.05, 0.1, 0.15) and explore how it affects the outputs of the model. The membership function of the steady state mean for each species is computed and illustrated in Fig 8, which clearly shows the uncertainty distribution of the steady state mean for each species. For example, the steady state mean for *G* can be at least about 109, while at most about 259 (at *α* level 0.0), and the most possible value is about 226 (at *α* level 1.0). For other species, we can obtain similar results. If we shorten the uncertainty interval of the parameter *k*_6_, e.g., *k*_6_ = (0.08, 0.1, 0.12), the range of the steady state mean for *G* will be shortened too, which is illustrated in Fig 9.

**Fig 10:**
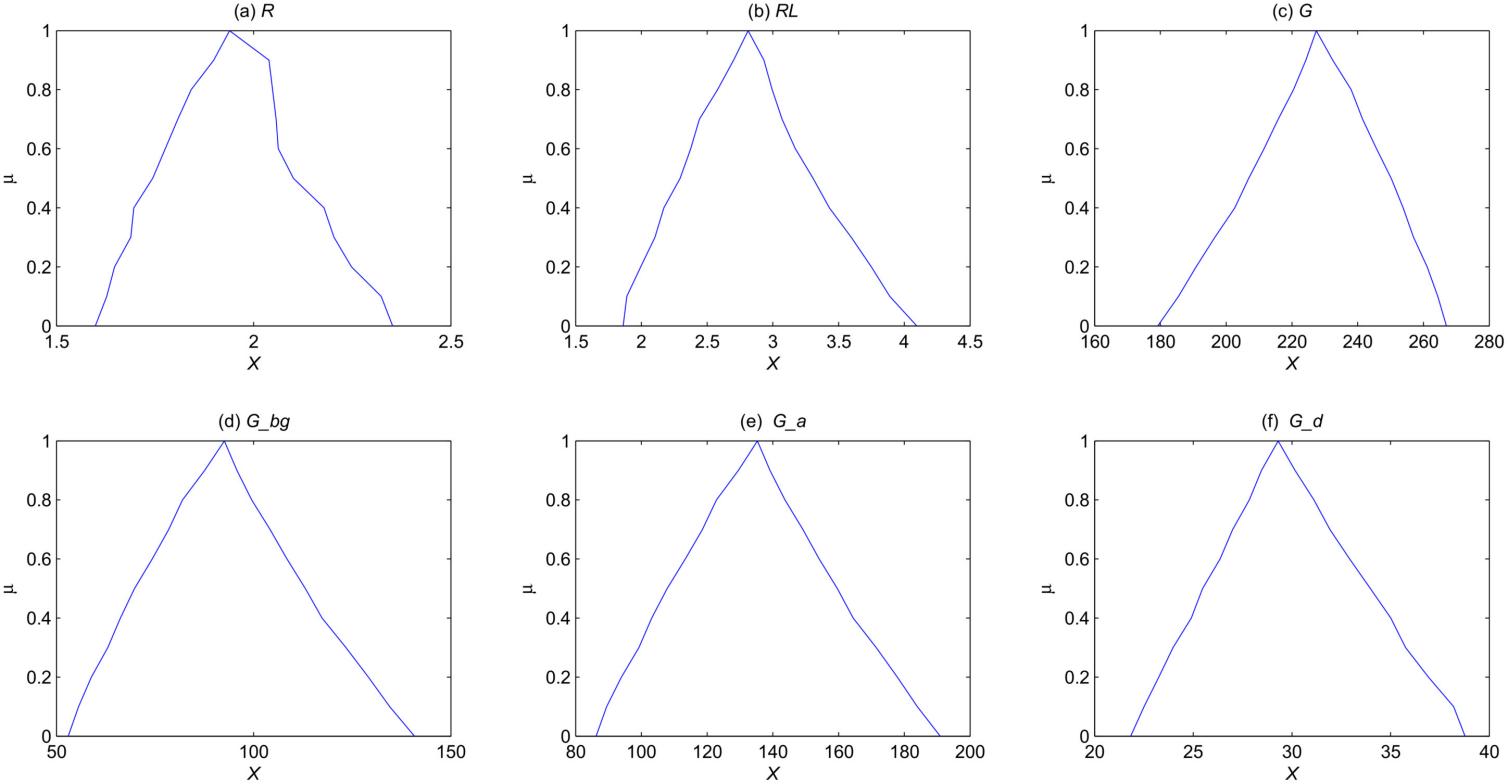
The membership function of the steady state state mean for each species of the yeast polarization model in the setting of two fuzzy parameters *k*_6_ = (0.08, 0.1, 0.12) and *k*_8_ = (10, 13.21, 15).

We then consider two fuzzy parameters, *k*_6_ = (0.08, 0.1, 0.12) and *k*_8_ = (10, 13.21, 15), and study how they affect the outputs of the model. The membership function of the steady state mean for each species is computed and illustrated in Fig 10. Of course, we can consider more fuzzy parameters, and we will obtain similar results as given above.

Now, let’s go back to the scenario discussed in the Introduction to see how to take advantage of our approach. If there exist qualitative as well as quantitative experimental finding for a biological phenomenon, each of which may describe different aspects of the system, we could either use stochastic Petri nets to describe the quantitative findings, with the kinetic parameters to be determined by parameter estimation methods, or use qualitative Petri nets to represent the qualitative findings, e.g., the biochemical reactions of the system with unknown parameters. If we assign fuzzy values to these unknown parameters with the help of biologists providing the required insights, we could combine the stochastic and qualitative Petri net models to form a more detailed model and then study it from a quantitative point of view. On the other hand, if some parameters naturally vary, e.g., between different individuals, and we want to see the effect of these variabilities on the outputs, we could also use fuzzy sets to describe the variabilities of parameters and use our approach for its study.

## Discussions and Conclusion

Taking into account the fact that in biological systems some kinetic parameters may be uncertain due to incomplete, vague or missing kinetic data, or naturally vary, e.g., between different individuals, experimental conditions, etc., we apply FSPNs by combining the strength of SPNs to model stochastic systems with the strength of fuzzy sets to deal with uncertain information.

We introduced a simulation-based analysis method and its implementation to simulate and analyze our FSPNs. Simulation-based analyzis techniques enjoy a couple of advantages. Most importantly, the size of the state space does not influence the efficiency of the approach; they work equally well for finite, but very large or even infinite state spaces. They also allow us to explore both transient and steady state behavior, are able to cope with non-markovian stochastic models, and can easily be adjusted to deal with various types of fuzzy numbers (beyond triangular-shaped ones). FSPNs combined with simulation provide us with a powerful tool to conveniently model and analyze biological systems with uncertain kinetic parameters.

We demonstrated the application of FSPNs and our analysis method using the yeast polarization model as case study. In summary, our approach is useful to integrate qualitative experimental findings into a quantitative model and to explore the system under study from the quantitative point of view. FSPNs provide a good means to consider parameter uncertainties in a model and to efficiently analyze how uncertain parameters affect the outputs of a model.

The way to deal with uncertain kinetic parameters and the simulation-based analysis method given in the paper equally apply to all quantitative modeling paradigms, i.e., also to continuous and hybrid Petri nets [46]. In a next step, we will consider more case studies which could take advantage by combining quantitative Petri nets with fuzzy sets.
